# Evaluating the impacts on health outcomes of Welsh Government funded schemes designed to improve the energy efficiency of the homes of low income households.

**DOI:** 10.23889/ijpds.v1i1.328

**Published:** 2017-04-19

**Authors:** Sian Morrison-Rees, Sarah Lowe, Martin Heaven

**Affiliations:** 1 Swansea University; 2 Welsh Government

## Background

Living in a cold and/or damp house is known to increase the risk of morbidity, mortality and excess winter deaths. To reduce fuel poverty and its adverse health effects in Wales the Welsh Government developed programmes to improve the energy efficiency of homes. These included the Home Energy Efficiency Scheme (HEES) and subsequent Warm Homes NEST scheme. Both schemes supported those most likely to be affected by fuel poverty, including low income and vulnerable households from 2000 to 2015. The energy efficiency measures provided included insulation and heating upgrades, such as a more efficient boiler.

## Aim

The overall aim of the project is to evaluate the health impacts of Welsh Government funded schemes designed to improve the energy efficiency of the homes of low income households through the use of existing data (HEES and NEST linked to the routine health records held in the SAIL Databank at Swansea University).

## Methods

A longitudinal dataset was created using the anonymised residential dwelling that has received home energy efficiency improvements linked to a summary of their health measures (hospital admissions, reason for the admission, GP prescriptions and clinical diagnoses).

Cohorts will be constructed of people who have already received the intervention to compare those who have not yet received the intervention but who went on to receive it two years later. We will use a stepped wedge design to construct an intervention and control cohort for each year of the study period. We will apply statistical techniques to conduct difference in difference (DID) estimations. This will allow us to compare any changes in the health of people before and after the intervention with any concurrent change in health in those who may require, but have yet to receive, the intervention.

## Results

We anticipate concluding the analysis in June in order for the results to inform the redesign of the successor scheme to Warm Homes NEST, due to be consulted on during 2016 and in place from April 2017. Our results will compare specific interventions for their impacts on health. We will show whether particular population groups e.g. those suffering from particular health condition, gain particular benefit from interventions.

## Conclusion

Our findings have the potential to inform more effectively focussed home energy efficiency schemes in order to reduce the numbers of people living in fuel poverty and thus improve the health and wellbeing of people living in Wales.

